# An Impossible Journey? The Development of *Plasmodium falciparum* NF54 in *Culex quinquefasciatus*

**DOI:** 10.1371/journal.pone.0063387

**Published:** 2013-05-03

**Authors:** Julia Knöckel, Alvaro Molina-Cruz, Elizabeth Fischer, Olga Muratova, Ashley Haile, Carolina Barillas-Mury, Louis H. Miller

**Affiliations:** 1 Laboratory of Malaria and Vector Research, National Institute of Allergy and Infectious Diseases, National Institutes of Health, Rockville, Maryland, United States of America; 2 Electron Microscopy Unit, Research Technologies Branch, Rocky Mountain Laboratories, National Institutes of Allergy and Infectious Diseases, National Institutes of Health, Hamilton, Montana, United States of America; 3 Laboratory of Malaria Immunology and Vaccinology, National Institute of Allergy and Infectious Diseases, National Institutes of Health, Rockville, Maryland, United States of America; Centro de Pesquisas René Rachou, Brazil

## Abstract

Although *Anopheles* mosquitoes are the vectors for human *Plasmodium* spp., there are also other mosquito species–among them culicines (*Culex* spp., *Aedes* spp.)–present in malaria-endemic areas. Culicine mosquitoes transmit arboviruses and filarial worms to humans and are vectors for avian *Plasmodium* spp., but have never been observed to transmit human *Plasmodium* spp. When ingested by a culicine mosquito, parasites could either face an environment that does not allow development due to biologic incompatibility or be actively killed by the mosquito’s immune system. In the latter case, the molecular mechanism of killing must be sufficiently powerful that *Plasmodium* is not able to overcome it. To investigate how human malaria parasites develop in culicine mosquitoes, we infected *Culex quinquefasciatus* with *Plasmodium falciparum* NF54 and monitored development of parasites in the blood bolus and midgut epithelium at different time points. Our results reveal that ookinetes develop in the midgut lumen of *C. quinquefasciatus* in slightly lower numbers than in *Anopheles gambiae* G3. After 30 hours, parasites have invaded the midgut and can be observed on the basal side of the midgut epithelium by confocal and transmission electron microscopy. Very few of the parasites in *C. quinquefasciatus* are alive, most of them are lysed. Eight days after the mosquito’s blood meal, no oocysts can be found in *C. quinquefasciatus*. Our results suggest that the mosquito immune system could be involved in parasite killing early in development after ookinetes have crossed the midgut epithelium and come in contact with the mosquito hemolymph.

## Introduction

Malaria, one of the most devastating human parasitic diseases, leads to ∼1 million deaths each year. About 50% of the world’s population live in areas where malaria is endemic and are therefore at risk of infection [1].

The vectors of human malaria parasites are female *Anopheles* spp. mosquitoes. When ingested during a blood meal, male and female gametocytes are “activated” and transform into male and female gametes. They fuse and form a zygote, which then develops into a motile ookinete. The ookinete crosses the mosquito midgut epithelium, reaches the basal side of the epithelium, and develops into an oocyst, lying in the space between the basal membrane of the epithelial cell and the basal lamina. Sporozoites develop inside the oocyst and are released after approximately ten days into the mosquito hemolymph. They invade the salivary glands and are transmitted to a new host during blood feeding of the mosquito.

In malaria-endemic areas, other mosquito species are also present, among them culicines (*Culex* spp., *Aedes* spp.). Culicine mosquitoes are–like anophelines–important vectors for human disease, transmitting filaria and several arboviruses. Additionally, culicine mosquitoes transmit avian *Plasmodium* species to birds [2]–[4]. *Culex quinquefasciatus* is a member of the *Culex pipiens* complex, the latter showing a worldwide distribution [4]. *C. quinquefasciatus* mosquitoes have been shown to be a major vector for West Nile [5], Rift Valley fever [6], [7], and St. Louis and Japanese encephalitis [8], [9] viruses as well as filarial worms to humans [4]. They are opportunistic feeders and rely on a variety of different hosts, showing great regional variability and ranging from humans to other mammals, birds, and sometimes reptiles and amphibians [10]–[13]. Although a number of studies confirm that *C. quinquefasciatus* feed on humans in malaria-endemic areas [12], [14], they have never been observed to transmit human *Plasmodium* spp. The same is true for other culicine mosquitoes. What causes the lack of parasite transmission is not known.

Several studies on field-caught as well as laboratory strains of different culicine mosquitoes revealed the presence of oocysts and sometimes even sporozoites after infection with human and other primate, mammalian, and avian malaria parasites. Among others, oocysts have been observed in *Mansonia uniformis* infected with *Plasmodium cynomolgi* as well as with *Plasmodium falciparum* [15], [16] and in *Culex bitaeniorhynchus* after infection with *P. falciparum* [17], although another study finds this mosquito refractory to *P. falciparum* [18]. Observation of sporozoites was very rare and with one exception [16] did not lead to infection of the salivary glands, but it clearly demonstrates that partial development of mammalian parasites can take place in culicine mosquitoes.

In *Anopheles gambiae*, several immune pathways and molecules are activated upon infection with *Plasmodium* spp. A major component of the anti-plasmodial response is the thioester-binding protein (TEP1)-complex, consisting of TEP1 [19]–[21] and two leucine-rich repeat (LRR) proteins: LRIM1 (leucine-rich repeat immune protein 1) and APL1 (*Anopheles*-*Plasmodium*-responsive leucine-rich repeat 1). These proteins, present in the mosquito hemolymph, mediate lysis of ookinetes after crossing of the midgut epithelium. [19], [22]–[24]. Recent work has demonstrated that protein nitration during midgut traversal makes *P. berghei* parasites detectable to the mosquito immune system and TEP1 [25]. TEP1 most efficiently eliminates *P. berghei*, but also mediates lysis of *P. falciparum* parasites in the *A. gambiae* Keele strain [26]–[28] and is involved in melanization of some *P. falciparum* clones in the *An. gambiae* L3-5 strain [29].

Several other major immune pathways have been shown to influence infection intensities, with the TOLL-pathway acting against *P. berghei* and the Imd-pathway being activated upon infection with *P. falciparum*, both targeting ookinetes early after invasion of the midgut epithelium [23], [26], [27], [30], [31]. The JAK-STAT pathway is active in *P. berghei* as well as *P. falciparum* infected mosquitoes and targets early oocysts [32].

Several genetic loci have been associated with higher susceptibility or refractoriness to *Plasmodium infection* in *An. gambiae* [33], [34] that contain a number of the above mentioned immune pathway genes. But despite the presence of powerful defense mechanisms, in susceptible anopheline mosquitoes, parasite numbers are only reduced and not completely eliminated, allowing parasite transmission to occur. In contrast, in culicine mosquitoes no transmission has been observed, but it is not known where and how parasites die. Understanding the mechanisms underlying the blockage of parasite transmission in a non-vector mosquito will provide insights into the requirements for successful development of the parasite in the human vector mosquito.

In this study, we investigate development of *P. falciparum* in *C. quinquefasciatus*. We show that ookinetes develop in the blood meal and are able to cross the midgut epithelium, but when they reach the basal side of the midgut, the parasites die.

## Materials and Methods

### Ethics Statement

The present study was carried out in strict accordance with the recommendations in the Guide for the Care and Use of Laboratory Animals of the National Institutes of Health. All animal procedures were approved by the National Institutes of Health Animal Care and Use Committee (ACUC, Protocol ID: LMVR102).

### Mosquito Rearing and Infection with *P. falciparum* NF54

Experiments were performed using *An. gambiae* G3 (MR4) and *C. quinquefasciatus* (Bamako) mosquito strains. The *C. quinquefasciatus* colony was established at NIH in 2005 from blood-fed and gravid females collected inside houses in Point G, Bamako, Mali. Mosquitoes are raised at 27°C and 75% relative humidity in a 13 hours light/11 hours dark cycle, and adult mosquitoes were maintained on a 10% Karo syrup solution and blood fed on chickens to maintain the colony.

For infection with *P. falciparum* NF54 parasites, 4- to 9-day-old female mosquitoes were fed by artificial membrane feeding. *P. falciparum* NF54 parasites were maintained in human O^+^ erythrocytes and RPMI 1640 medium supplemented with 25 mM HEPES, 50 mg/ml hypoxanthine, 25 mM NaHCO_3_, and 10% (v/v) heat-inactivated human serum type O^+^ [35]–[37]. Blood constituents were purchased from Interstate Blood Bank Inc., Memphis, TN. USA. Gametocytogenesis was induced as described [38], and 14- to 16-day-old mature gametocyte cultures (stage V) were fed to mosquitoes through parafilm-covered, prewarmed (37°C) membrane feeders for 30 minutes.

Midguts were dissected 8–11 days post blood feeding and stained with 0.1% mercurochrome in dH_2_O for ten minutes. To determine infection intensities, oocysts were counted by light microscopy.

### Immunostaining of Parasites in Mosquito Blood Meals

Individual midguts were dissected 20 and 30 hours after the membrane feed in a drop of PBS and the blood meal washed out of the midgut epithelium. The blood meal was resuspended in 2–3 µl of PBS and spread in a defined field of 1 cm × 1 cm for *An. gambiae* and 1 cm × 1.5 cm for *C. quinquefasciatus*. Slides were dried for at least ten minutes and then stored at 4°C in a sealed bag with DriRite prior to immunostaining.

For immunostaining parasites in the blood meals, slides were warmed to room temperature (RT) for 45 minutes, removed from the bag, and fixed in ice-cold 90% acetone, 10% methanol for ten minutes at –20°C. Slides were dried and washed in PBS for five minutes at RT and subsequently blocked in 5% milk in PBS for ten minutes. Blocking solution was removed and the slides washed in wash buffer (PBS, 0.01% Tween 20) for five minutes, shaking at RT. Slides were then incubated with mouse-anti-Pfs25 antibody (4B7, 1∶1000 in PBS, 1% BSA) in a humidified chamber for one hour and washed three times for ten minutes in wash buffer. The slides were placed in the humidified chamber and incubated with secondary Alexa Fluor® 555 goat anti-mouse IgG (Life Technologies, Gaithersburg, Maryland, USA) at 1∶1000 in PBS, 1% BSA for one hour in the dark. Slides were washed two times for ten minutes in wash buffer and once for ten minutes in PBS. To seal the slides, a small drop of Vectashield hardset with DAPI was added and the slide covered with a cover slip. Slides were stored in the dark at 4°C until parasites were counted using a fluorescence microscope.

### Determination of Ookinete Conversion Rate and Total Parasite Numbers in Mosquito Blood Meals

To determine the fraction of parasites that successfully transformed into ookinetes (ookinete conversion rate), 100 parasites were counted in each immunostained blood meal smear. Round forms (gametes or zygotes) and ookinetes were counted separately, and the percentage of each of the two developmental stages was calculated. Retort forms were included in the gamete counts, and only fully developed ookinetes were counted as such.To determine the total number of parasites, ookinetes were counted in 25 fields of 0.1 mm × 0.1 mm for each slide by fluorescence microscopy and subsequently extrapolated to the entire area of the field of 100 mm^2^ and 150 mm^2^ for *An. gambiae* and *C. quinquefasciatus*, respectively.

### Determination of Blood Meal Size of *An. gambiae* and *C.* *quinquefasciatus*

The blood meal size of *An. gambiae* and *C. quinquefasciatus* was determined by hemoglobinometry [39], [40]. Hemoglobin is converted into the stable cyanomethemoglobin, which has maximum absorbance at 540 nm. Color intensity at this wavelength is proportional to the total hemoglobin content of the sample. Briefly, female mosquitoes were fed by membrane feeding using uninfected human red blood cells at 40% hematocrit (40% uninfected red blood cells, 60% human serum). Blood-fed mosquitoes were individually flash frozen in liquid N_2_ at different time points after the blood meal. To the tubes, 500 µl of Drabkin’s reagent (Sigma-Aldrich, St. Louis, Missouri, USA) was added, and the mosquitoes were thoroughly ground and incubated for at least 30 minutes at room temperature. The tubes were vortexed and centrifuged at 5000×*g* for five minutes. Supernatant (300 µl) was transferred to a new tube containing 800 µl dH_2_O for dilution purposes. 300 µl of the diluted reaction mix were then transferred to a 96-well plate and absorption read at 540 nm using a Synergy HT spectrophotometer (BioTek, Winooski, Vermont, USA).

A calibration curve was created for each experiment using known volumes of the same blood mixture used for the feeding (1, 2, 3, 4, 5, 6, and 7 µl) to determine the total amount of blood ingested by the mosquito. Additionally, five unfed mosquitoes were collected in each experiment and were processed the same way as the blood-fed females. The average absorption was used as a correction factor for the absorption obtained for blood-fed mosquitoes.

### Immunostaining of Parasites in the Mosquito Midgut Epithelium

To determine the number of parasites in the midgut epithelium, midguts were dissected 30 hours after the blood feeding in cold Ashburner’s PBS. Midguts were fixed in 4% paraformaldehyde in Ashburner’s PBS for 30 seconds and the midgut contents were removed. The epithelial tissue was fixed in 4% paraformaldehyde in Ashburner’s PBS for one hour at RT or kept in 1% paraformaldehyde shaking at 4°C for longer storage. Midguts were washed three times for five minutes in Ashburner’s PBS and blocked for one to two hours in PBT (1× Ashburner’s PBS, 1% BSA, 0.1% Triton X-100 for *An. gambiae* and 0.25% Triton X-100 for *C. quinquefasciatus*). The midgut tissue was incubated overnight at 4°C with an anti-Pfs25-monoclonal antibody (4B7, 1∶1000 in PBT) and washed three times for 20 minutes in PBT, followed by incubation with the secondary antibody (1∶1000 in PBT) for three hours (Alexa Fluor® 555 goat anti-mouse IgG; Life Technologies). The tissue was washed two times for 20 minutes with PBT and once for 20 minutes with PBS. Subsequently, midguts were incubated with Phalloidin AlexaFluor® 488 (1∶50 in PBS, 1% BSA) (Life Technologies) to stain the actin. Tissue was mounted on slides in Vectashield with DAPI (Vector Laboratories, Inc., Burlingame, Connecticut, USA) and stored in the dark at 4°C. Parasites were counted using a DEMIRE 2 epifluorescence microscope (Leica, Solms, Germany), and parasites were localized in the tissue using a TCS SP2 confocal microscope (Leica). Images derived from epifluorescence microscopy were processed using Adobe Photoshop 6.0 (Adobe Systems Inc, San José, California, USA), and confocal images were processed using Huygens Essentials (Scientific Volume Imaging, Hilversum, The Netherlands) and Imaris 7.5.2 (Bitplane, South Windsor, Connecticut, USA) software.

### Transmission Electron Microscopy (TEM)

*An. gambiae* and *C. quinquefasciatus* midguts were dissected 24 hours after a *P. falciparum*-infected blood meal and fixed overnight at 4°C with 2.5% glutaraldehyde, 4% paraformaldehyde in 0.1 M sodium cacodylate buffer, pH 7.4. Samples were post-fixed one hour with 0.5% osmium tetroxide, 0.8% potassium ferricyanide, one hour with 1% tannic acid, and overnight with 1% uranyl acetate at 4°C. Samples were dehydrated with a graded ethanol series and embedded in Spurr’s resin. Thin sections were cut with a Leica UCT ultramicrotome (Vienna, Austria) stained with 1% uranyl acetate and Reynold’s lead citrate prior to viewing at 120 kV on a BioTwin Spirit transmission electron microscope (FEI Tecnai, Hillsboro, Oregon, USA). Digital images were acquired with an AMT digital camera system (AMT, Chazy, New York, USA) and processed using Adobe Photoshop 6.0.

### Statistical Analyses

**Figure 1:**
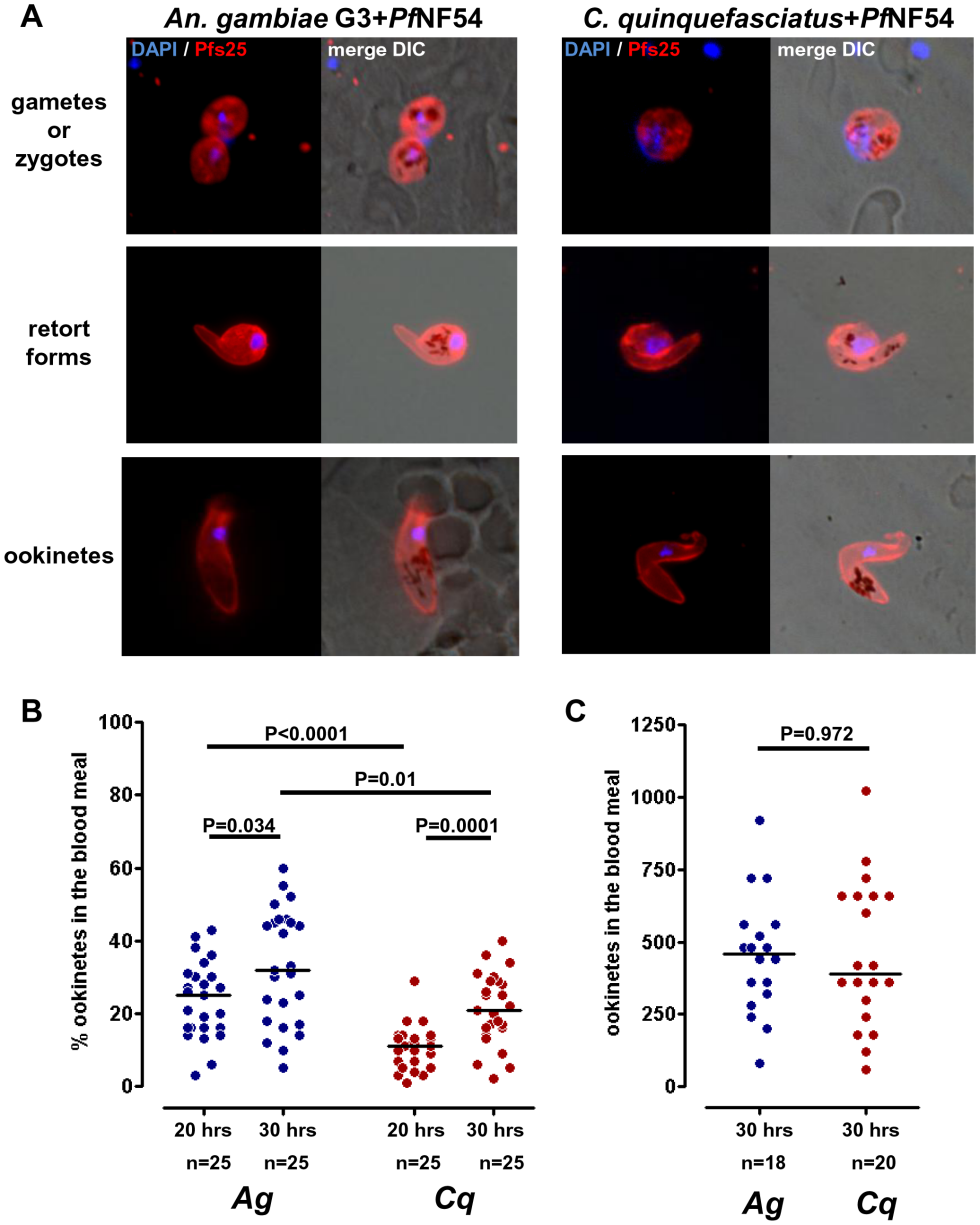
Development of *Plasmodium falciparum* NF54 in the mosquito blood meal. (A) Epifluorescence images of parasite stages observed in the blood meal of *Anopheles gambiae* G3 (left panel) and *Culex quinquefasciatus* (right panel) 20 hours after the mosquitoes were fed on a *P. falciparum* NF54 gametocyte culture. Immunostaining was done using a monoclonal anti-Pfs25 antibody to stain the parasites (red) and DAPI to visualize the nuclei (blue). (B) Ookinete conversion rate at 20 and 30 hours after the mosquito blood meal. One hundred parasites were counted for each sample and the percentage of ookinetes calculated. Each dot represents the percentage of ookinetes in one blood meal and the lines are the medians for all samples. Three independent experiments were performed and the combined data from the three infections is shown here. The groups were compared using a Mann-Whitney U test. P-values for each comparison are indicated in the graph. (C) Total number of ookinetes in the mosquito blood meal 30 hours after infection. Each dot represents the number of ookinetes found in a given blood meal, the medians are indicated as lines. Two independent experiments were performed and the combined data is shown here. Similarity was tested using a Mann-Whitney U test, which revealed that the two groups are not significantly different (P = 0.972).

For statistical analyses of the ookinete conversion rate (Figure 1B), data from three independent experiments were combined. Because some samples could not be used for parasite counting (blood meals tend to flake off the glass slide during the staining procedure and parasites could not be counted), not all groups in each experiment had the same number of samples. For combination of the data, the group with the lowest sample number from each experiment was taken in full. The data for each of the remaining groups was randomized using Microsoft Excel 2010 software and equal numbers of samples from those groups were taken after randomization and combined with the data of the two other feeds (6 samples each were taken from Exp. #1, 9 from #2 and 10 from #3). To compare the number of ookinetes in the blood meals of *An. gambiae* and *C. quinquefasciatus* (Figure 1C), data from two independent feeds were combined. The medians were compared using the Mann-Whitney U test and Graph Pad Prism 5 (GraphPad Software, Inc., La Jolla, California, USA).

For analyses of parasite numbers in the midgut epithelium at 30 hours and 8 days, data from all five infections were combined and analyzed using the van Elteren test and SAS 9.3 software (SAS Institute Inc, Cary, North Carolina, USA).

## Results

### *P. falciparum* NF54 Ookinetes Develop in the Blood Bolus of *C. quinquefasciatus*

We dissected mosquitoes 20 and 30 hours after a *P. falciparum* NF54-infected blood meal to investigate whether ookinetes can develop in the midgut lumen of *C. quinquefasciatus*. The blood bolus was extracted from the midgut epithelium, and blood meal smears were immunostained using an antibody targeting the ookinete surface protein Pfs25. As shown in Figure 1A, three forms of parasites are present in both *An. gambiae* and *C. quinquefasciatus* blood meals after infection of the mosquito. Round parasite forms are either female macrogametes or zygotes. Because expression of the targeted protein, Pfs25, starts in female gametes and continues until the early oocyst stage [41], macrogametes and zygotes cannot be distinguished with the antibody used in this study; however, retort forms as well as banana-shaped ookinetes can be observed, clearly showing that fertilization has taken place and ookinetes are able to develop in the blood meal of *C. quinquefasciatus*.

We were primarily interested in the efficacy of the development of mature ookinetes in the blood meal, since these parasite stages are the ones subsequently invading the midgut epithelium. We performed three independent feeding experiments and the combined data from all experiments is presented in Figure 1B. To determine the ookinete conversion rate, we counted a total of 100 parasites per blood meal and determined the percentage of ookinetes compared to round and retort forms combined.

As shown in Figure 1B, twenty hours after the blood feeding of the mosquito, in *An. gambiae* 25% of parasites are ookinetes and the proportion slightly increases to 32% at thirty hours (P = 0.034, Mann-Whitey U test). In *C. quinquefasciatus*, the percentage of mature ookinetes is only 11% at twenty hours, significantly less than in *An. gambiae* at this time point (P<0.0001, Mann-Whitney U test). However, the proportion of ookinetes increases significantly and reaches 21% thirty hours after the blood feeding (P = 0.0001, Mann-Whitney U test). The proportion of ookinetes is still significantly lower in *C. quinquefasciatus* than it is in *An. gambiae* thirty hours after the blood feeding (P = 0.01, Mann-Whitney U test). This could be an indication that the development of ookinetes in *C. quinquefasciatus* is less efficient than in *An. gambiae*. However, this result could also have been caused by differential loss of parasites in the blood meal, due to differences in digestion, the gut flora of the mosquito, or other factors.

For this reason we also determined the total number of ookinetes that are present in the blood meal thirty hours after the blood feeding. This would give us a better idea how many parasites are present in the blood bolus that are able to invade the midgut epithelium at this time point.

We counted the number of ookinetes in whole blood meals from two experiments which gave good infections in the *An. gambiae* control (8 day median >5 oocysts per midgut in *An. gambiae*) and combined the data from the two feeds for better statistical analyses. Generally, we observed a large variation of ookinete numbers between individual mosquitoes, for *An. gambiae* as well as for *C. quinquefasciatus.* Numbers range from approximately 100 ookinetes in one blood meal to over 1000 in another mosquito that had been fed with the same gametocyte culture (Figure 1C). Variations in parasite numbers are similar in both mosquitoes and the medians of 460 and 390 ookinetes per midgut are not significantly different between *An. gambiae* and *C. quinquefasciatus* (P = 0.972, Mann-Whitney U test).

Our results clearly show that thirty hours after the blood meal the number of mature ookinetes that are present in *C. quinquefasciatus* is only 15% lower than in *An. gambiae*. Those parasites are in principle able to invade the midgut epithelium. Even though zygote to ookinete transition might be compromised in *C. quinquefasciatus* to some extent, development of *P. falciparum* in the mosquito midgut lumen is not the limiting step for the observed lack of transmission.

### Blood Meal Size in An. gambiae and *C. quinquefasciatus* is Similar, but Digestion in *C. quinquefasciatus* is Slower

**Figure 2:**
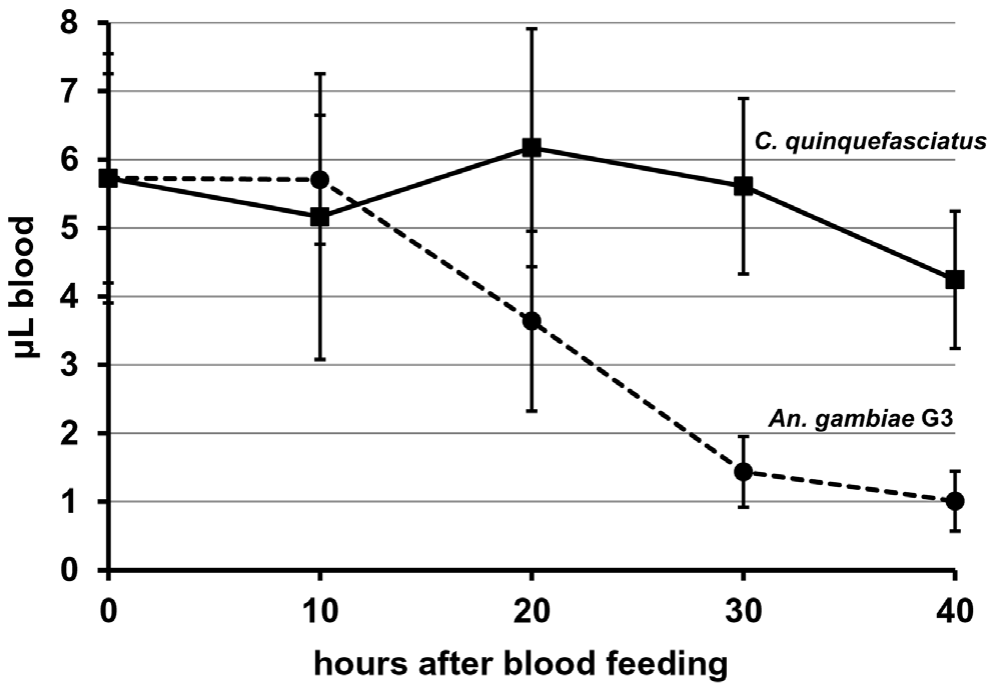
Comparison of blood intake of *Anopheles gambiae* and *Culex quinquefasciatus* during a blood meal. Total hemoglobin content of female mosquitoes fed on a 40% hematocrit blood solution was determined by hemoglobinometry at different time points after a blood meal. Ten mosquitoes were analyzed for each time point and the average amount of ingested blood calculated using a standard curve. Values are shown as mean ± standard deviation. The volume of blood corresponding to the determined hemoglobin amount was compared between *An. gambiae* (--•--) and *C. quinquefasciatus* ( —▪— ).

The amount of ingested blood determines the number of gametocytes that reach the midgut of the mosquito and subsequently can fertilize and develop into ookinetes. Additionally, the rate of digestion can influence parasite development and survival in the midgut lumen. The total hemoglobin content of blood-fed female *An. gambiae* and *C. quinquefasciatus* mosquitoes was measured at different time points after a blood meal to determine the volume of blood ingested and the rate of digestion of the blood meal. As shown in Figure 2, our hemoglobinometry analyses reveal that the amount of hemoglobin immediately after feeding is similar in both mosquito species and corresponds to a total volume of 5.7±1.53 µl blood in *An. gambiae* and 5.7±1.82 µl blood in *C. quinquefasciatus*. The number of ingested parasites should thus be the same in *An. gambiae* and *C. quinquefasciatus*.

The concentration of hemoglobin stays constant in *An. gambiae* and *C. quinquefasciatus* for the following ten hours; then digestion progresses faster in *An. gambiae*. After ten hours, the blood meal decreases steadily until minimal hemoglobin remains 40 hours post blood feed. In contrast, in *C. quinquefasciatus*, the hemoglobin remains at the same level for 30 hours and has only decreased by 30% after 40 hours. This indicates that digestion of the blood meal progresses slower in *C. quinquefasciatus* compared to *An. gambiae* and could affect parasite development.

### *P. falciparum* NF54 Parasites are Lysed in the Midgut Epithelium

In susceptible *Anopheles spp*. mosquitoes, motile ookinetes that develop in the blood meal cross the midgut epithelium, reaching the basal lamina, where they develop into oocysts. We dissected mosquito midguts thirty hours after the mosquito blood meal to determine whether ookinetes or oocysts can be found on the basal side of the midgut epithelium in *C. quinquefasciatus.* The midgut epithelium was stained with an antibody against Pfs25 to visualize the parasites.

**Figure 3:**
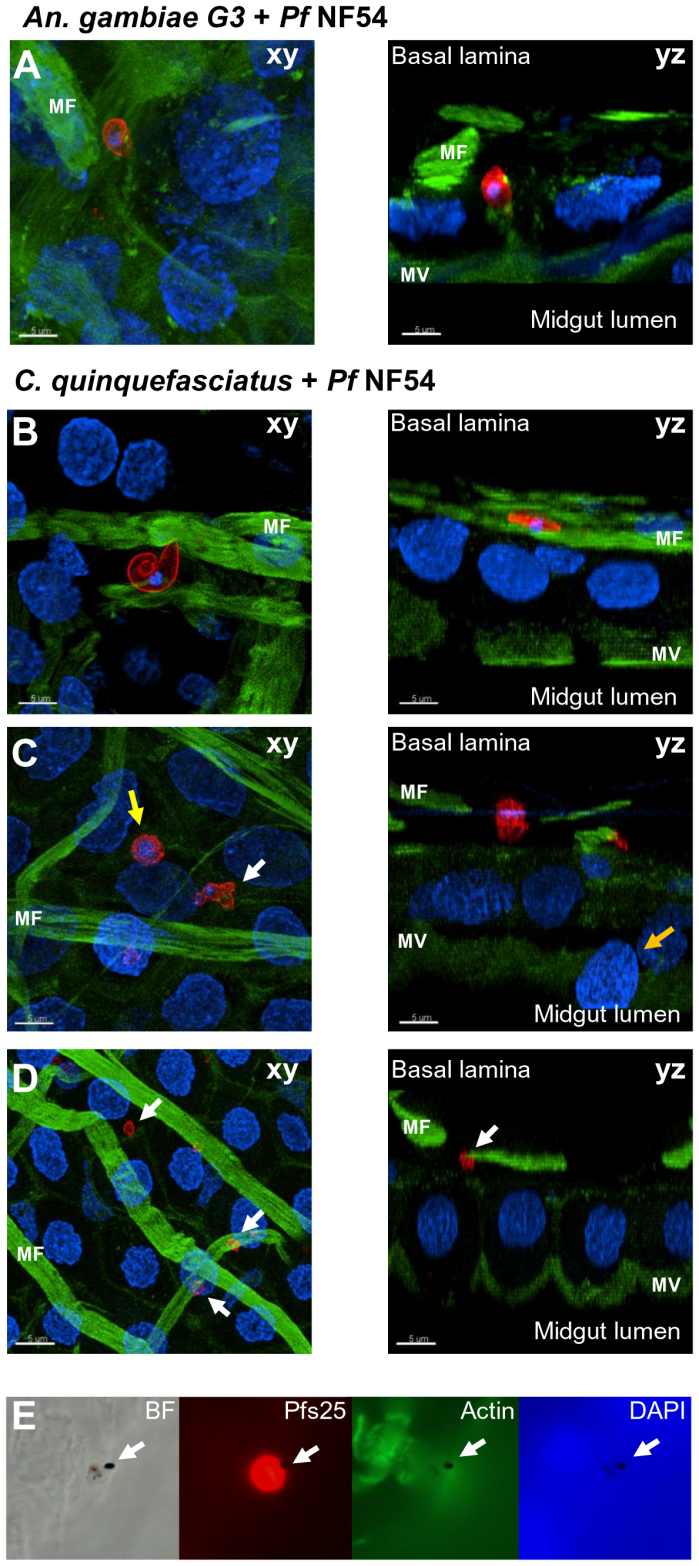
Confocal imaging of parasites in the mosquito midgut epithelium. Midgut epithelial tissue was collected 30 hours after the mosquito blood meal. (A) *Plasmodium falciparum* NF54 parasite in *Anopheles gambiae* G3. (B–D) *Culex quinquefasciatus* midgut epithelium containing (B) a live *P. falciparum* parasite and (C, D) parasites in different stages of lysis. Parasites in (C) and (D) have lost their even rim staining, which now appears dotted. Some parasites still contain nuclei (C, yellow and white arrow), but most parasites do not contain nuclei anymore (D, white arrows). A midgut cell is “budding off” into the midgut lumen (C, orange arrow). Shown is a section of the z-stack in the location of the parasite (left) and a side view of the midgut epithelium to localize the parasites (right). (E) Epifluorescence imaging of a parasite in *C. quinquefasciatus*. Note the black pigment associated with the parasite, which is visible in all fluorescent channels (arrows). Parasites were stained with a monoclonal anti-Pfs25 antibody (red), actin was stained using Phalloidin (green), and nuclei were visualized with DAPI (blue). MF: Muscle fibers on the basal side of the midgut; MV: microvilli. The scale bar indicates 5 µm.

In the positive control–*An. gambiae* infected with *P. falciparum* NF54–ookinetes and young oocysts can be observed on the basal side of the midgut epithelium (Figure 3A, Figure S1 A-E). In *C. quinquefasciatus*, we also find ookinetes that appear normal (Figure 3B, Figure S1 H). Some parasites are round, indicating they started to transition into early oocyst stage (Figure S1 I). However, the observation of healthy appearing parasites is very rare in *C. quinquefasciatus*. In contrast to *An. gambiae*, many of the midguts contain parasites that are lysing. Figure 3C shows a parasite in the midgut epithelium of *C. quinquefasciatus* that resembles a young oocyst (yellow arrow). Parasites have also been seen in the more elongated banana-shape characteristic for ookinetes (Figure S1 K). In contrast to the even rim staining of Pfs25 in *An. gambiae*, in *C. quinquefasciatus* the parasite staining appears uneven and dotted. In some parasites, the nuclear staining is prominent inside the parasite (Figure 3C; xy, yellow arrow). However, the majority of parasites lack a nucleus. Parasite staining most of the times appears as single circles or accumulations of small circles, some with DNA staining, others without (Figure 3C, D, white arrows, Figure S1 L). Circular shapes with even or dotted rims stained with Pfs25 are smaller than parasites observed in *An. gambiae* and have diameters of 1.64±0.2 (*n* = 23) to 2.58±0.34 µm (*n* = 25). Only a few of the larger sized circles were observed to contain nuclei.

Another observation we made in our microscopy studies is that the parasites in the *C. quinquefasciatus* midgut epithelium with Pfs25 staining often have black particles that appear to be associated with the parasite (Figure 3E, arrows). The black areas are visible in the fluorescent channels, which is unusual and cannot be seen in live parasites. Healthy parasites contain hemozoin crystals, which are characteristic for *P. falciparum* parasites but can only be seen in brightfield or DIC images, not in the fluorescent channels. When imaged in the microscope’s brightfield channel, the black staining observed in the lysing parasites appears sometimes to colocalize with the hemozoin crystals; therefore, it could be hemozoin that is later released from the lysing parasites. Another possibility is that it is melanin deposited by the mosquito on the parasites as part of the killing process. Melanized parasites remain visible on the mosquito midgut and would be detectable at 8–10 days after the infection, as seen in the *Plasmodium*-refractory *An. gambiae* L3-5 strain that melanizes parasites [42]. However, eight days after an infected feed, no melanized parasites can be found in *C. quinquefasciatus*. We therefore do not know what the black substance is, although it seems to appear as part of parasite degradation and is cleared by the mosquito.

### Parasites are Localized on the Basal Side of the Midgut Epithelium

To investigate whether the parasites we observed in *C. quinquefasciatus* were able to cross the midgut epithelium of *C. quinquefasciatus*, we analyzed side views of the confocal z-stacks. To identify the location of the parasites, midgut cells were stained with Phalloidin to visualize actin. Phalloidin strongly stains the muscle fibers (Figure 3, MF) surrounding the midgut outside the basal lamina, marking the basal side, and the microvilli (Figure 3, MV), located on the apical side of the midgut cells. In Figure 3, B–D (right panel), live parasites as well as the remnants of lysing parasites are located in close proximity to the muscle fibers that surround the epithelial cells and are located above the basal lamina. This observation clearly shows that parasites are able to invade the midgut epithelial layer by 30 hours after the infection and reach the basal side of the midgut. In these samples we cannot determine if parasites are located inside the midgut cell or outside between the basal membrane of the midgut cell and the basal lamina. We only see they have traveled to the basal side of the cell, but the parasites could still be located inside, unable to exit the cell they invaded.

**Figure 4:**
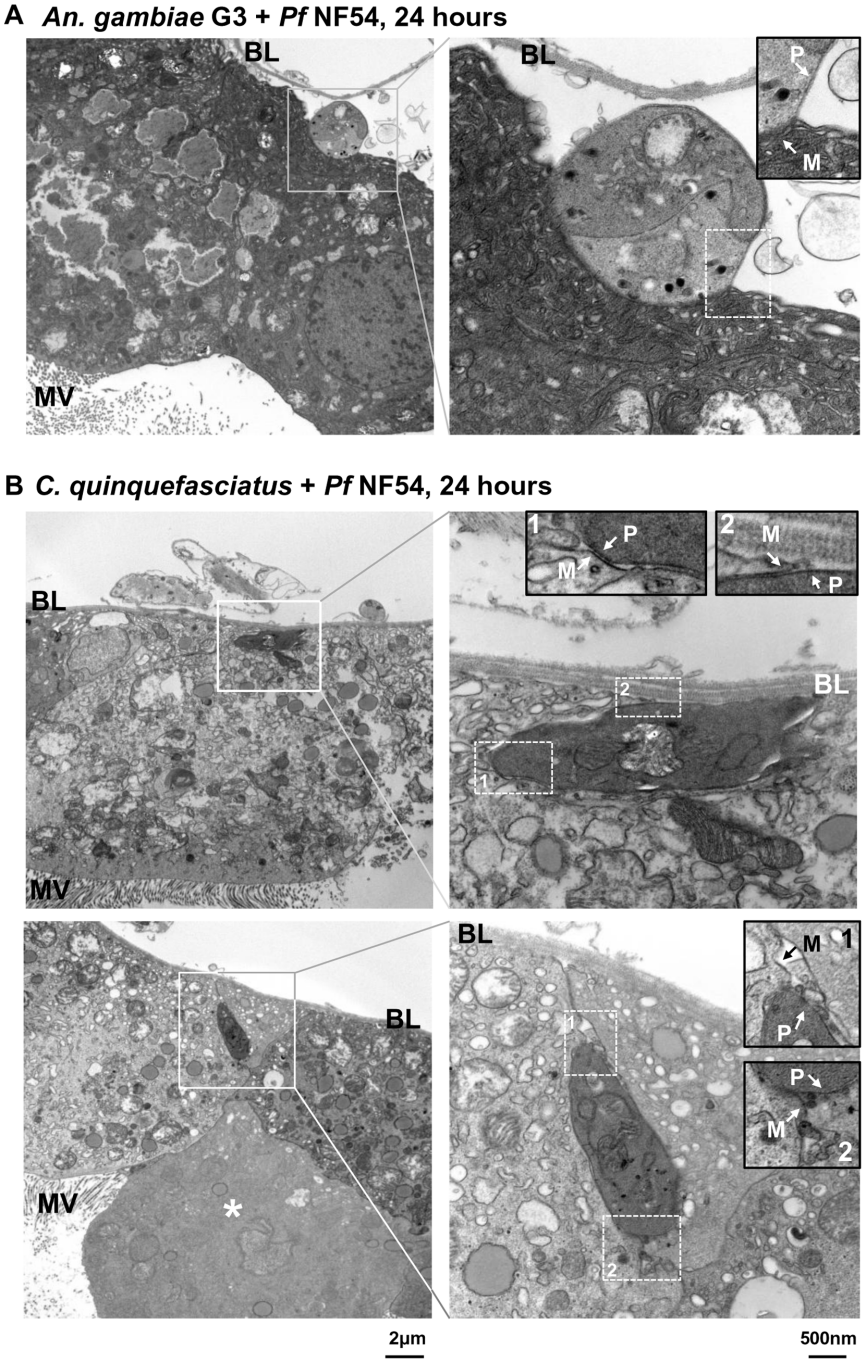
Transmission electron microscopy of infected midguts 24 hours post feed on *Plasmodium falciparum* NF54-infected blood. Shown are overviews including the entire midgut epithelial layer (left) and magnifications of the parasite (right). (A) Parasite in the midgut epithelium of *Anopheles gambiae* G3, which is located on the basal side of the midgut epithelium underneath the basal lamina. (B) *P. falciparum* NF54 in the midgut of *Culex quinquefasciatus*. Parasites are located on the basal side of the midgut epithelium (overview left panel) outside the midgut cells. Parasite 1 (top) is located underneath the basal lamina outside the midgut cell, as it is surrounded by two membranes, one belonging to a midgut cell (arrow, M) and one of parasite origin (arrow, P) (see insets in right panel). The organelles inside the parasite are less pronounced than in *An. gambiae* (A), indicating lysis of the parasite (right panel). Parasite 2 (bottom) is located between two adjacent midgut cells toward the basal side of the epithelium. Two membranes can be seen (right panel inset, arrows M, P), showing an extracellular location of the parasite. Note here that one midgut epithelial cell (asterisk) is not connected to the basal lamina and lacks microvilli and most organelles, indicating apoptosis. MV: microvilli; BL: Basal lamina.

Transmission electron microscopy (TEM) of parasites in the midgut epithelium was performed 24 hours after the mosquito blood meal to visualize more precisely the subcellular localization of the parasites in the midgut epithelium. Figure 4A shows a parasite in the midgut epithelium of *An. gambiae*. The parasite is located in the space between the basal membrane of the midgut epithelial cells and the basal lamina (Figure 4A, inset, BL), which seems to be detached from the cells. The parasite crossed the midgut cells and reached the basal side, the place where parasites develop into oocysts.

In contrast to *An. gambiae*, in the *C. quinquefasciatus* midgut epithelium *P. falciparum* parasites appear to be disintegrating and missing most of the characteristic organelles (Figure 4B), indicating that the two parasites shown are lysing. The parasites are clearly located on the basal side of the midgut. One parasite is located on the basal side directly underneath the basal lamina (Figure 4B, top), whereas the second is located between two adjacent midgut cells (Figure 4B, bottom). Two membranes are present surrounding the parasites, of which one seems to belong to a midgut cell (Figure 4, insets, M) and the other to the parasite (Figure 4, insets, P). Ookinetes possess a double membrane. However, the resolution of our images does not allow us to clearly distinguish the inner and outer parasite membranes. In both samples, the inner of the two membranes surrounds the parasite, whereas the outer one follows the boundaries of the midgut cell, indicating that both parasites are located outside the midgut cell, one close to the basal lamina and the second one between two midgut cells.

### Most Parasites are Lysed in *C. quinquefasciatus*

**Figure 5:**
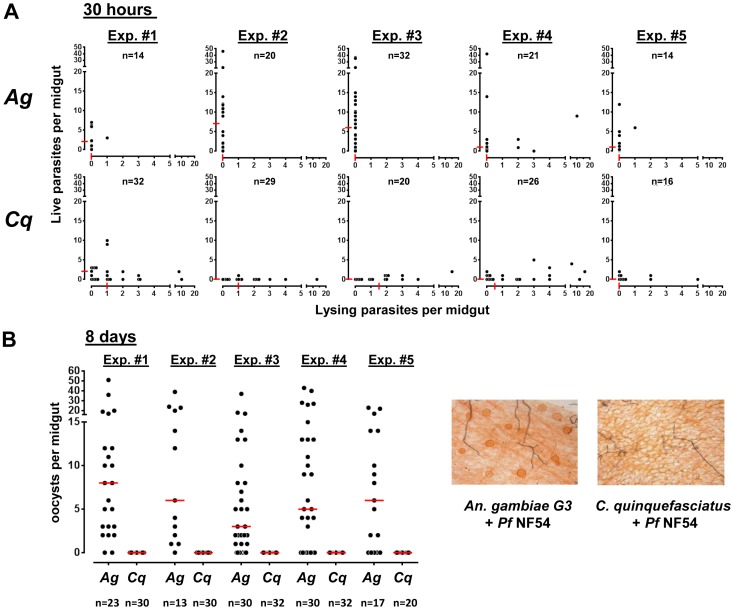
Parasite development in the mosquito midgut epithelium. (A) Number of live vs. lysing parasites in the midgut epithelium of *An. gambiae* (top) and *C. quinquefasciatus* (bottom) 30 hours after a *Plasmodium falciparum*-infected blood meal. Parasites were stained with a monoclonal anti-Pfs25 antibody and counted using a DEMIRE 2 Epifluorescence microscope. The results of five independent infections are shown here (Exp. #1 - #5). Each dot represents one midgut and the number of live vs. lysing parasites in the given midgut. The medians are given as red lines on the axes of the graphs and the sample size (n) is indicated for each group. Infection intensities in *An. gambiae* and *C. quinquefasciatus* were compared combining data from the five experiments and using the van Elteren test and were significantly lower in *C. quinquefasciatus* compared to the *An. gambiae* control (P<0.0001). (B) Number of oocysts found on the midgut epithelium eight days after the infection in five independent infections (same as for 30 hours). Midguts were stained with mercurochrome and oocysts counted in a light microscope at 40× magnification. Infection intensities were compared between *An. gambiae* and *C. quinquefasciatus*. The median oocyst number in *An. gambiae* was 6 oocysts per midgut, whereas in *C. quinquefasciatus* no oocysts could be found (P<0.0001, van Elteren test). (C) Representative images of mercurochrome stained mosquito midguts 8 days after *P. falciparum* NF54 infection. The *Anopheles gambiae* midgut contains oocysts (orange circles), whereas in *Culex quinquefasciatus* no oocysts can be found.

To determine how many live and lysing parasites are present in the midguts of *C. quinquefasciatus* compared to *An. gambiae*, we counted the parasites in five independent experiments thirty hours after an infectious blood feeding (Figure 5A). In *An. gambiae*, we mainly observe live parasites, only a few midguts contained some lysing parasites. Medians of live parasites range from one to seven parasites per midgut. In contrast, midguts of *C. quinquefasciatus* mainly contained lysing parasites and only a few live parasites could be seen. Taking into account the results from all five infection experiments, we find significantly fewer live parasites in the midgut epithelium of *C. quinquefasciatus* than in *An. gambiae* (P<0.0001, van Elteren test). The main difference we observed here is the presence of lysed parasites in *C. quinquefasciatus*, which we rarely find in *An. gambiae* mosquitoes (Figure 5A).

To investigate if the few live parasites we observe in *C. quinquefasciatus* at thirty hours developed into oocysts, we also analyzed merchurochrome stained midguts 8 days after the infection with *P. falciparum* NF54. No oocysts are found in *C. quinquefasciatus* at this time point, whereas in *An. gambiae,* most of the midguts are infected and contain oocysts (Figure 5B; P<0.0001; van Elteren-test).

### Damage of the Midgut Epithelium in *C. quinquefasciatus* after Parasite Invasion

It has been shown in different mosquito-parasite systems that parasite invasion causes massive epithelium damage and leads to the death of the invaded cell. We analyzed the integrity of the midgut epithelial cells of *An. gambiae* and *C. quinquefasciatus* after infection with *P. falciparum* NF54 parasites by confocal and electron microscopy. In our immunostained midgut samples we observed midgut cells extruding toward the midgut lumen in both, *An. gambiae* and *C. quinquefasciatus* (Figure 3C, yz, orange arrow, Figure S1 D, L, arrows), but these events were very rare. In *C. quinquefasciatus,* we found a damaged midgut cell close to an ookinete, in which the nucleus is fragmented and the DNA is condensed (Figure S1 H, arrows). The cell is still fully integrated in the epithelial layer, but the disintegration of the DNA is indicative of apoptosis, possibly caused by parasite invasion. Additionally, in one of our TEM images we observed a midgut cell in *C. quinquefasciatus* showing signs of apoptosis such as loss of microvilli and organelles (Figure 4B, bottom, asterisk). The cell is no longer connected to the basal lamina and is protruding into the midgut lumen. However, in both mosquito species, the majority of epithelial cells in close proximity to ookinetes that had reached the basal side of the midgut were intact, and exhibited no obvious signs of cell damage (Figure 3A, B and D, Figure S1 A-C, H, I). Cells were organized in a single layer, the nuclei were all in the same plane, in the middle of the cell and had intact microvilli. In *An. gambiae*, we noticed that some midgut cells near a parasite had an unusual shape. Instead of the regular “honeycomb” pattern in which the cells are usually organized (Figure S1 G), groups of cells showed a “flower-like” organization with one part of the cells stretched to the center (Figure S1 F, arrows). This suggests that a damaged cell had been expelled from the epithelium and the surrounding cells are stretching to fill in the space to keep the integrity of the midgut epithelial layer.

## Discussion

The main vectors for human malaria parasites are female *Anopheles* mosquitoes. When ingested by the mosquito during a blood meal, parasites develop and eventually sporozoites are transmitted and infect another human during the mosquito’s next blood meal. In contrast, *Culex* spp. mosquitoes have not been observed to transmit malaria to humans. Here, we investigate the ability of *P. falciparum* NF54 to develop in *C. quinquefasciatus*. Some *Plasmodium* species, such as *P. berghei*, fail to form ookinetes in *Culex* mosquitoes [43]. However, our analyses of the blood meals of *P. falciparum*-infected mosquitoes revealed that the number of mature ookinetes that are present in the blood bolus of *C. quinquefasciatus* thirty hours after the blood feeding is only 15% lower than in *An. gambiae*. Therefore, development of *P. falciparum* in the midgut lumen is not severely affected and is not the limiting factor for parasite transmission in *C. quinquefasciatus*.

Recently published work by Baton and Ranford-Cartwright [44] shows that *P. falciparum* 3D7 ookinetes develop in the blood meal of *Anopheles albimanus*, but fail to invade the midgut epithelium. This is in contrast to our findings, since in *C. quinquefasciatus* we observe *P. falciparum* ookinetes on the basal side of the midgut, showing that at least a small fraction of the parasites from the blood bolus invaded the midgut epithelium. This is consistent with other studies, in which oocysts of *P. falciparum* were found in different culicine mosquitoes [15], [16], [45]. Oocyst development requires ookinete development and midgut invasion and therefore both events must have taken place in these mosquitoes.

Comparison of the total numbers of ookinetes present in the blood meal of *An. gambiae* and *C. quinquefasciatus* to the numbers of developing parasites on the basal side of the midgut epithelium reveals that egress of parasites from the midgut lumen is relatively inefficient and accounts for big losses in parasite numbers in both mosquito species. Even in *An. gambiae*, the number of ookinetes in the blood meal is about 50 times higher (Figure 1C) than the median number of parasites at thirty hours post infection (Figure 3A), showing that only a small fraction of ookinetes actually invade the midgut epithelium. Similar observations have been made in *An. stephensi* infected with *P. falciparum* 3D7 [44].

The peritrophic matrix (PM) is the first physical barrier that parasites must overcome before they can invade the midgut epithelium. The PM is a chitineous membrane formed during blood meal digestion around the blood bolus to protect the midgut epithelium from bacteria [46]. Because we clearly observe that parasites are able to invade the midgut of *C. quinquefasciatus* mosquitoes, they must be able to cross the PM, but we do not know with what efficiency.

Ookinete invasion inflicts damage to the mosquito midgut, and different repair mechanisms that are activated to ensure the integrity of the epithelium have been described in other mosquito-parasite systems. One of them involves “budding off” of damaged cells into the midgut lumen [47]–[49] and a second one the formation of “actin cones” [50]. In our microscopy studies, we found a few midgut cells that are extruding into the midgut lumen or show signs of apoptosis both in *An. gambiae* and *C. quinquefasciatus*. This may indicate that in both mosquitoes at least a portion of the parasites take an intracellular route when they cross the epithelium. We observed several examples of damaged cells “budding off” from the midgut, but found no evidence of “actin cone” formation.

In *C. quinquefasciatus* parasites die shortly after they reach the basal side of the midgut epithelium. We observed only a small number of live parasites thirty hours after the mosquito was infected. At this time most parasites were already dead and undergoing extensive lysis, indicated by uneven *Pfs25* surface staining, loss of organelles and enucleated parasites. We were unable to find any mature *P. falciparum* oocysts in *C. quinquefasciatus*, in contrast to other studies in different culicine mosquitoes, in which oocysts were observed [15]–[17]. The presence of lysing parasites on the basal side of the midgut epithelium and the absence of oocysts later on clearly indicates that the final losses of parasites in *C. quinquefasciatus* take place early after invasion of the midgut.

On the basal side of the midgut epithelium, ookinetes need to interact with the laminin and other components in the basal lamina of the mosquito to form oocysts in *Anopheles* [51]–[53], and likely also require other signals coming from the mosquito environment to proceed in their development into oocysts. Those triggers could be missing in *C. quinquefasciatus* mosquitoes and lead to parasite death, although it has been demonstrated in several non-vector insects that *Plasmodium* ookinetes can develop when they are injected directly into the hemolymph, bypassing the midgut epithelium [54]–[56]. Based on these findings, and our observations of young oocyst stage parasites on the basal side of the midgut in our confocal studies and a parasite that seems to be in close contact with the basal lamina, seen in TEM, it seems unlikely that a lack of receptors or signaling events cause the destruction of parasites we observe for *P. falciparum* NF54 in *C. quinquefasciatus*, although it cannot be absolutely excluded.

A major challenge the parasite faces after exiting the midgut cell is exposure to the mosquito hemolymph. The hemolymph contains antiparasitic effector molecules produced by the mosquito immune system to fight infection, resulting in either lysis or melanization of pathogens. We sometimes find black particles associated with the *P. falciparum* NF54 parasites on the basal side of the midgut epithelium in *C. quinquefasciatus*. This could be the beginning of a melanization reaction to eliminate the parasites. However, we do not observe fully melanized parasites on the mosquito midgut eight days after the infection, so the exact nature of the black substance remains unclear. The parasites we observe lyse on the basal side of the midgut epithelium or on exposure to the mosquito hemolymph. This could be an indication that the mosquito immune system, at least to some extent, is involved in parasite death.

However, most anopheline species, like culicine mosquitoes, do not transmit human malaria parasites. The main reason for this is maybe that most mosquitoes do not feed on humans and never come in contact with the parasite. But even among all the mosquitoes that feed on humans and therefore could transmit the parasite, there are significant differences in vector competence for a given parasite. So the question is what makes a good (competent) vector? Or the other way around, what is it that abolishes parasite transmission in most mosquitoes compared to a few that can harbor human *Plasmodium*? Could there be a gene that is so powerful that it eliminates all parasites? We show that *P. falciparum* NF54 parasites are able to develop in the blood meal and invade the midgut epithelium of *C. quinquefasciatus*. The 3D7A clone, originally derived from NF54, can form ookinetes in *An. albimanus*, but parasites remain in the midgut lumen [44]. These differences suggest that there will not be one single mechanism that eliminates human *Plasmodium* in all non-vector mosquitoes. Additionally, one must take into account the parasite, and its ability to adapt to its vector. Only recently it has been shown that some African strains of *P. falciparum* are able to completely evade the TEP1 immune response in the *An. gambiae* L3-5 strain that efficiently melanizes *P. falciparum* isolates of American and Asian origin [29]. These observations give a great example of the complexity of vector and parasite interactions required for transmission. The parasite has to overcome many obstacles before successful transmission occurs. In our case, the infection of *C. quinquefasciatus* with *P. falciparum* NF54, the fact that we observe lysing parasites on the basal side of the midgut, opens the possibility that mosquito immune factors could be involved in parasite death. Further studies of the immune response of *C. quinquefasciatus* to *P. falciparum* NF54 infection is needed to elucidate this possibility.

## Supporting Information

Figure S1 **Confocal imaging of parasites in the mosquito midgut epithelium 30 hours post** ***P. falciparum*** **NF54 infection.** Panels (A)-(F) show parasites in the midgut epithelium of *An. gambiae* G3. (A) An ookinete invading the midgut epithelium and reaching the basal side. (B), (C) Oocysts developing on the basal side of the midgut. (D) A parasite in the midgut epithelium. One midgut cell that was most likely damaged by parasite invasion is expelled into the midgut lumen. The nucleus is protruding towards the luminal side and located more apical than the nuclei of the surrounding midgut cells (arrow). (E) A young oocyst on the basal side of the midgut epithelium. The epithelial cells seem to be intact (yz), but a group of cells close to a parasite show a flower-like pattern (F) with one side of the cell extending towards the others (arrows). This is an indication for midgut repair after a damaged cell got expelled into the midgut lumen and the adjacent midgut cells stretch to fill in the space and ensure integrity of the midgut epithelium. The section of the midgut epithelium was taken in the central region of the midgut cells invaded by the parasite shown in (E). The asterisk marks the location of the parasite on the basal side. (G) A section of the midgut epithelium of *An. gambiae* 30 hours after an uninfected blood meal. Compare the regular “honeycomb” pattern of the cells to the “flower-like” shape caused by parasite invasion shown in (F). Panels (H)-(L) show parasites in the midgut epithelium of *C. quinquefasciatus*. (H) An ookinete on the basal side of the midgut epithelium. In one midgut cell close to the parasite, the nucleus appears to be condensed (arrow), which could be a sign of apoptosis after damage of the cell due to invasion of the parasite. (I) An oocyst and (K) an ookinete on the basal side of the midgut epithelium of *C. quinquefasciatus.* The surface staining is uneven, indicating that both parasites are lysing. (L) Two parasites located on the basal side of the midgut. A midgut cell close to the parasite on the bottom seems to be extruding from the midgut epithelium (xz, arrow). Shown are sections of the z-stacks in the location of the parasite (xy) and a side view of the midgut epithelium (xz or yz) to localize the parasites. The parasites were stained with a monoclonal anti-Pfs25 antibody (red), actin was stained using Phalloidin (green), and nuclei were visualized with DAPI (blue). MF: Muscle fibers on the basal side of the midgut; MV: microvilli. The scale bar indicates 5 µm.
(TIF)
