# Supplementary figures and images for: An Impossible Journey? The Development of Plasmodium falciparum NF54 in Culex quinquefasciatus
Source: PLoS One. 2013 May 3;8(5):e63387. doi: 10.1371/journal.pone.0063387 (PMC3643899; doi:10.1371/journal.pone.0063387)

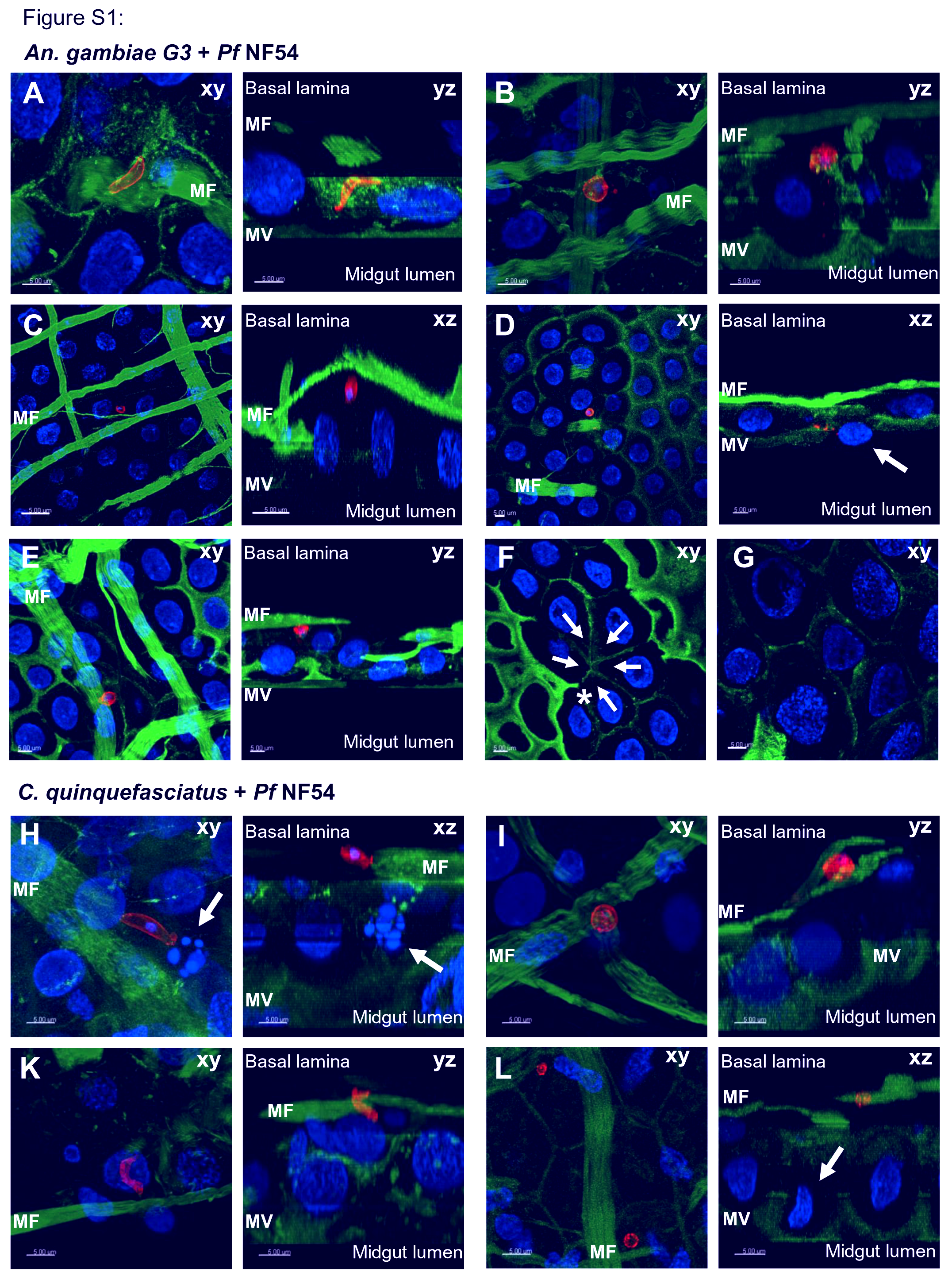

Supplement: Figure S1 — Confocal imaging of parasites in the mosquito midgut epithelium 30 hours post P. falciparum NF54 infection. Panels (A)-(F) show parasites in the midgut epithelium of An. gambiae G3. (A) An ookinete invading the midgut epithelium and reaching the basal side. (B), (C) Oocysts developing on the basal side of the midgut. (D) A parasite in the midgut epithelium. One midgut cell that was most likely damaged by parasite invasion is expelled into the midgut lumen. The nucleus is protruding towards the luminal side and located more apical than the nuclei of the surrounding midgut cells (arrow). (E) A young oocyst on the basal side of the midgut epithelium. The epithelial cells seem to be intact (yz), but a group of cells close to a parasite show a flower-like pattern (F) with one side of the cell extending towards the others (arrows). This is an indication for midgut repair after a damaged cell got expelled into the midgut lumen and the adjacent midgut cells stretch to fill in the space and ensure integrity of the midgut epithelium. The section of the midgut epithelium was taken in the central region of the midgut cells invaded by the parasite shown in (E). The asterisk marks the location of the parasite on the basal side. (G) A section of the midgut epithelium of An. gambiae 30 hours after an uninfected blood meal. Compare the regular “honeycomb” pattern of the cells to the “flower-like” shape caused by parasite invasion shown in (F). Panels (H)-(L) show parasites in the midgut epithelium of C. quinquefasciatus. (H) An ookinete on the basal side of the midgut epithelium. In one midgut cell close to the parasite, the nucleus appears to be condensed (arrow), which could be a sign of apoptosis after damage of the cell due to invasion of the parasite. (I) An oocyst and (K) an ookinete on the basal side of the midgut epithelium of C. quinquefasciatus. The surface staining is uneven, indicating that both parasites are lysing. (L) Two parasites located on the basal side of the mid [file pone.0063387.s001.tif]
